# Immunosuppression by piperine as a regulator of the NLRP3 inflammasome through MAPK/NF-κB in monosodium urate-induced rat gouty arthritis

**DOI:** 10.14202/vetworld.2022.288-298

**Published:** 2022-02-11

**Authors:** Galih Aji Kuncoro Jati, Nazzun Assihhah, Anas Ardiana Wati, Siti Isrina Oktavia Salasia

**Affiliations:** 1 Department of Clinical Pathology, Faculty of Veterinary Medicine, Universitas Gadjah Mada, Yogyakarta, Indonesia; 2 Department of Pharmaceutical Chemistry, Faculty of Pharmacy, Universitas Gadjah Mada, Yogyakarta, Indonesia

**Keywords:** gout, immunosuppression, monosodium urate, NLRP3 inflammasome, piperine

## Abstract

**Background and Aim:**

Gouty arthritis is a metabolic disorder involving monosodium urate (MSU) crystal deposition as a key initiator of acute inflammation. Dysregulation of the nucleotide-binding oligomerization domain, leucine-rich repeat, and pyrin domain-containing protein 3 (NLRP3) inflammasome is associated with the pathogenesis of gout through the maturation of interleukin-1β. Piperine (PIP) is a phytochemical with an anti-inflammatory activity that has the potential as an alternative treatment for gout. In this study, we examined the effect of PIP in immunosuppression of gout inflammation through the regulation of the NLRP3 inflammasome.

**Materials and Methods:**

An *in silico* study was done by pharmacodynamic modeling of PIP in suppressing MSU-induced inflammation through disruption of the NLRP3 inflammasome. *In vivo* tests, including inflammatory assessment, histopathology, cytology, estimation of lipid peroxidation index, and detection of systemic inflammatory reactants, were performed on two groups using preventive and curative protocols.

**Results:**

*In silico* studies of molecular docking demonstrated the activity of PIP as a competitive inhibitor of the mitogen-activated protein kinases/nuclear factor-kappa B axis, upstream of the NLRP3 inflammasome. Analysis of gout models with curative and preventive protocols revealed the immunosuppression activity of PIP by reducing inflammatory symptoms, inhibiting tophus formation resulting from NETosis, reducing cartilage erosion, inhibiting leukocyte exudation, suppressing lipid peroxidation index, and inhibiting the production of C-reactive protein.

**Conclusion:**

The results demonstrate the activity of PIP as an immunosuppressant in gout flare. These findings indicate the potential of PIP as a candidate for prophylactic and therapeutic agent for the treatment of gouty arthritis.

## Introduction

Gouty arthritis is characterized by hyperuricemia and the deposition of monosodium urate (MSU) crystals in articular and periarticular tissues in the extremities, which can trigger acute inflammation [1,2]. Acute gouty inflammation is characterized by pain, swelling from edema, and erythema that attacks the joints [3]. Based on the diagnosis and symptoms, the prevalence of gout is approximately 11.9-24.7% [4]. The incidence of gout increases and may be accompanied by comorbidities [5].

The pathophysiology of gout is a manifestation of the innate immune inflammatory response triggered by MSU microcrystals. MSU accumulation stimulates monocytes, macrophages, and neutrophils to secrete cytokines and chemokines, such as interleukin (IL)-1β, tumor necrosis factor-α (TNF-α), IL-8, IL-6, and monocyte chemotactic factor, which further induce and amplify the inflammatory cascade [6]. A previous study has shown that the production of inflammatory mediators by monocytes and macrophages as a result of MSU phagocytosis plays an important role in the initiation of gout pathogenesis [7]. During an acute gout attack, an influx of neutrophils into the tissues accompanies the secretion of pro-inflammatory cytokines by monocytes and macrophages. This results in the appearance of inflammatory symptoms at the onset of acute gout [8]. The nucleotide-binding oligomerization domain, leucine-rich repeat, and pyrin domain-containing protein 3 (NLRP3) inflammasome complex is a vital component of the innate immune regulators. NLRP3 inflammasome dysregulation enhances the pathogenesis of gout [9]. Martinon *et al*. [10] demonstrated the role of the NLRP3 inflammasome in MSU-induced IL-1β secretion. Activation of the NLRP3 inflammasome in MSU-induced gout involves two signals. The activation of this complex involves primary signaling through nuclear factor kappa B (NF-κB) that causes NLRP3 and pro-IL-1β mRNA expression [11,12]. Mitogen-activated protein kinases (MAPK) signaling pathways, including JNK/ERK/p38, activate NF-κB during the regulation of the NLRP3 inflammasome [13]. JNK-1 (MAPK family) also acts on the second signal through Ser198 of NLRP3 and Tyr146 of adapter apoptosis-associated speck-like protein containing a caspase-recruitment domain (ASC) [14,15]. Because of its important function, the MAPK/NF-κB axis is a potential target for the NLRP3 inflammasome regulation during gout.

Current medications used by gout patients include colchicine (Col) and nonsteroidal anti-inflammatory drugs (NSAIDs). Gout patients treated with NSAIDs can exhibit kidney intoxication, gastropathy, hyperkalemia, liver function abnormalities, and headaches [1]. Therefore, alternative treatments and anti-inflammatory therapeutic strategies are needed with minimal side effects to optimize the management of gout. Herbal medicines have become alternatives and generated interest in recent decades because of their potential activity and low toxicity [5]. Piperine (PIP) is a phytochemical compound of the *Piperaceae* family. PIP exhibits immunomodulator, hepatoprotective, anti-carcinogen, anti-inflammatory, anti-oxidant, and anti-microbial effects [16]. Bang *et al*. [17] reported that PIP has anti-inflammatory and anti-arthritis activity in synoviocytes stimulated with IL-1β. PIP inhibits the production of nitric oxide and TNF during its immunosuppressive effect [18]. In addition, PIP is known to inhibit inflammasome-induced LPS-ATP NLRP3 activity in the HK-2 epithelium [19].

This study aimed to evaluate the anti-inflammatory mechanism of PIP and the efficacy of PIP as an anti-gout agent through preventive and curative action in an MSU-induced gout rat model.

## Materials and Methods

### Ethical approval

All experimental procedures were approved by the Animal Care and Use Committee, Faculty of Veterinary Medicine, Universitas Gadjah Mada (No. 00075/EC-FKH/Int./2021).

### Study period and location

This study was carried out from June to September 2021 at the Clinical Pathology Laboratory of Faculty of Veterinary Medicine and Biochemistry Laboratory of Faculty of Medicine, Public Health, and Nursing, Universitas Gadjah Mada.

### Molecular docking of PIP

A computational approach in the form of molecular docking was made with the help of the 2015 Molecular Operating Environment (MOE) software (Chemical Computing Group, Montreal, Canada). The three-dimension (3D) structure of an inhibitor of nuclear factor-kappa B kinase beta subunit (IKK-β) (PDB ID: 2KIK) and c-Jun N-terminal kinases 1 (JNK-1) (PDB ID: 3PZE) were downloaded from the Protein Data Bank website (www.rcsb.org/pdb) [20]. Protein selection was based upon the resolution, source organism, and method of X-ray diffraction. The PIP compound was downloaded from PubChem (https://pubchem.ncbi.nlm.nih.gov/) [21]. Protein preparation, which was done before the docking process, included protonated 3D and energy minimization. Identifying the binding site was done with the site finder menu available in the MOE software. The affinity strength was obtained after the docking process between the ligand and the target protein was completed.

### MSU crystal preparation

A total of 800 mg of uric acid (C_5_H_4_N_4_O_3_) (Santa Cruz Biotechnology, Dallas, Texas) were dissolved in 155 mL of boiling water containing 5 mL of 1 N NaOH (Merck, Darmstadt, Germany). After adjusting the pH to 7.2, the solution was cooled for 24 h at 4°C. The MSU crystal precipitate was then evaporated and sterilized by heating at 120°C for 3 h. MSU was dissolved in 0.9% NaCl solution (Merck) at a concentration of 40 mg/mL before use. The MSU solution was then cooled for 3 days at 4°C before injection [22].

### Experimental protocol

For the *in vivo* experiment, two protocols were used. Protocol I, which was preventive, was designed to determine the potential of PIP as a prophylactic agent for gout. Protocol II was curative and determined the potential of PIP as a therapeutic agent for gout. For each protocol, four treatment groups (n=3) were used, a sham group with 1.25 mL of 0.9% NaCl solution, negative control with 1.25 mL (5 mg) MSU (40 mg MSU in 1 mL of 0.9% NaCl solution), treatment with MSU+PIP (100 mg/kg BW), and a positive control with MSU+Col (0.28 mg/kg BW). PIP (Nootropics, Grandville, USA) was dissolved in dimethyl sulfoxide (Merck,) at a 60 mg/mL concentration and Col (Pratapa Nirmala, Jakarta, Indonesia) was dissolved in distilled water at a concentration of 0.5 mg/mL. In protocol I, PIP and Col were administered orally every day at 9:00 AM for 3 days. MSU injection was performed 1 h following PIP and Col administration on day 3. Injection of NaCl solution in the sham group was done simultaneously with MSU. In protocol II, MSU and NaCl solution injection were carried out once at the beginning of the experiment at 8:00 AM, followed by oral PIP and Col 1 h after injection. Administration of PIP and Col in protocol II was done for 3 days. Injection of MSU and saline solution was done at the intra-plantar region of the left leg.

### *In vivo* gout induction

Twenty-four male Wistar rats (300±30 g) were divided into two protocols (curative and preventive protocols). Each protocol consists of four groups, namely sham, MSU, MSU+PIP, and MSU+Col (n=3 per group). Food was provided according to laboratory standards, and drinking water was available *ad libitum* for 20 days of the experiment. Acclimatization was carried out for 7 days before the experiment. The rats were placed in a room with a light-dark cycle of 12 h. Weighing was done before the treatment based on the experimental protocol. Before injection, the rats were first anesthetized with a mixture of 0.1 mL of ketamine (Agricareperu, Salamanca, Spain) (2 mg/mL) and 0.1 mL of distilled water. Injection of 1.25 mL of 0.9% NaCl solution (sham group) and 1.25 mL of MSU (MSU, MSU+PIP, MSU+Col group) was done in the intra-plantar region of the left leg.

### Inflammation assessment

The assessment of rat paw inflammation was performed by measuring the plantar circumference of the foot in the same position, using the tie line method, and scoring. Rat paw inflammation assessment was carried out 1 h before the injection of MSU and 0.9% NaCl solution. For protocol I, repeated measurements were made at 2, 4, 8, 12, and 24 h after injection. For protocol II, repeated measurements were made at 2, 4, 8, 12, 24, 48, and 72 h after injection. Measurements with the tie line method were carried out in triplicate. Percent edema was measured as swelling rate={(b/a)-1}×100%, where “a” was the circumference before MSU injection and “b” was the circumference after MSU injection [6]. Scoring was done macroscopically on a scale of 1-3, where 0=no inflammation, 1=mild inflammation 2=moderate inflammation, and 3=severe inflammation [23]. After the rat paw assessment, the blood was collected and centrifuged at 7000 g for 10 min using centrifuge PLC-01 (Gemmy Industrial, Taipei, Taiwan), and the serum was separated. The rats were then anesthetized using ketamine and euthanized by decapitation. Necropsy was done by taking plantar (part of the paw) samples of the right foot, liver, and spleen. Plantar samples were fixed in 10% formalin (Leica, Melbourne, Australia). In contrast, the liver and spleen were homogenized in phosphate-buffered saline (PBS, pH 7.2), then cooled at 4°C to prepare the tissue homogenate. Serum, foot samples, and homogenized supernatant were then further assessed for histological, cytological, and biochemical test.

### Histopathological observation

Rat right paws were fixed in 10% formalin were then prepared forhistological analysis by hematoxylin and eosin (H&E) staining (Leica, Melbourne, Australia). The fixed tissue was decalcified with 0.5M ethylenediaminetetraacetic acid, pH 7.4 (Sigma-Aldrich, Missouri, USA). The tissue was then embedded in paraffin (Leica, Melbourne, Australia) and cut to a thickness of 4 μm using a rotary microtome (Thermo Fisher, Massachusetts, USA). The slides were then stained with H&E. The histopathological changes of the tissues were then analyzed by light microscopy (Olympus, Tokyo, Japan) at 100x and 400x magnification and images were digitally captured at a resolution of 1360x1024 pixels with an Olympus DP70 digital camera (Olympus, Tokyo, Japan).

### Giemsa staining

The collected rat paw samples were then separated from the skin. An impression smear of plantar tissue was performed to determine the pattern of cell infiltration in the exudate following MSU-induced inflammation. The preparations were fixed with 50% methanol (Merck) and stained with Giemsa, 1:10/Giemsa (stock solution, Merck): PBS, pH 7.2. After 30 min, they were rinsed with distilled water and the infiltration pattern was observed by light microscopy (Olympus, Tokyo, Japan) at 100x and 400x magnification and images were digitally captured at a resolution of 1360x1024 pixels with an Olympus DP70 digital camera (Olympus, Tokyo, Japan).

### Determination of serum malondialdehyde (MDA)

For the MDA assay, 4 mL of thiobarbituric acid reagent (Merck) (20% acetic acid, 0.8% thiobarbituric acid) was added to 1 mL of the serum sample. The mixture was homogenized and incubated at 90°C for 80 min and then cooled at 4°C in an ice bath for 10 min. Then, 4 mL of butanol solution (Merck) were added to the mixture and centrifuged at 3000 x g for 15 min. The supernatant was collected, and the absorbance was read at 510 nm using a spectrophotometer (Thermo Fisher).

### Liver C-reactive protein (CRP) detection

The 10% liver homogenate supernatant was tested for CRP using CRP-latex. A total of 40 μL of the sample was dripped onto a card and mixed with 50 μL of CRP-latex reagent contains sodium azide 0.95 g/L (Glory Diagnostics, Barcelona, Spain). The mixture was placed on a mechanical rotator (Gemmy Industrial, Taipei, Taiwan) at 2.24 g for 2 min. A qualitative analysis of CRP was performed based on the degree of visible agglutination.

### Statistical analysis

Statistical analysis was performed using GraphPad Prism 9.0 software (GraphPad Software, San Diego, California). The tabulation results of the tie line method, scoring, and MDA data are presented as the mean±SD. The normal distribution of the data from samples (n=3) was evaluated using the Shapiro–Wilk test (α=0.05). Statistical significance was determined by one-way analysis of variance, followed by Tukey’s *post hoc* test for scoring data and Dunnet’s test for tie line data. p<0.05 was considered statistically significant. The inflammatory, histopathological, cytological, and CRP assessment data was carried out descriptively.

## Results

### PIP suppresses the activity of the NLRP3 inflammasome via the MAPK/NF-κB axis

**Figure-1:**
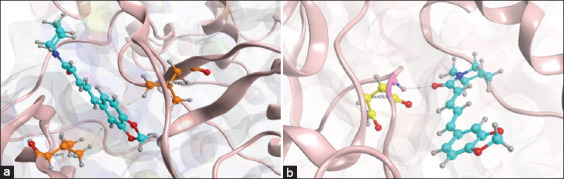
Model of competitive inhibition of piperine (PIP) on the components of the Mitogen-activated protein kinases/NF-B axis. (a) Visualization of the 3D PIP ligand interaction with the JNK-1 active site. (b) Visualization of the 3D PIP ligand interaction with the active site of inhibitor of nuclear factor-kappa B kinase beta subunit.

Modeling of the anti-inflammatory effect of PIP for the inhibition of the pathogenesis of MSU-induced gouty arthritis was done using an *in silico* study. The results of a molecular docking-based study demonstrated the action of PIP as a competitive inhibitor of the JNK-1 and IKK-β proteins (Figure-1). PIP interacts hydrophobically with the benzene group of residues Val 40 and Val 158 at the active site of JNK-1 (Figure-1a). Visualization of the *in silico* study also showed the presence of hydrogen bonds between the PIP carbonyl functional group and the amino acid residue, Asn 263, located at the IKK-β active site (Figure-1b). The PIP ligand had a root mean square deviation (RMSD) value of 0.8569Å against IKK-β and 0.6914Å against JNK-1.

The RMSD value represents a validation measure that shows the comparison value between the conformation of the cocrystal ligand and the computational ligand. Based on the molecular docking results, the PIP bond affinity values for IKK-β and JNK-1 were −5.7428 kcal/mol and −6.0626 kcal/mol, respectively.

### PIP reduces inflammation in a plantar of rat model of MSU-induced gout

Based on the pharmacodynamics of PIP as an anti-inflammatory that disrupts the NLRP3 inflammasome cascade, *in vivo* experiments were done to demonstrate the preventive (protocol I) and curative (protocol II) action of PIP on anti-arthritis gout resulting from MSU deposition.

**Figure-2:**
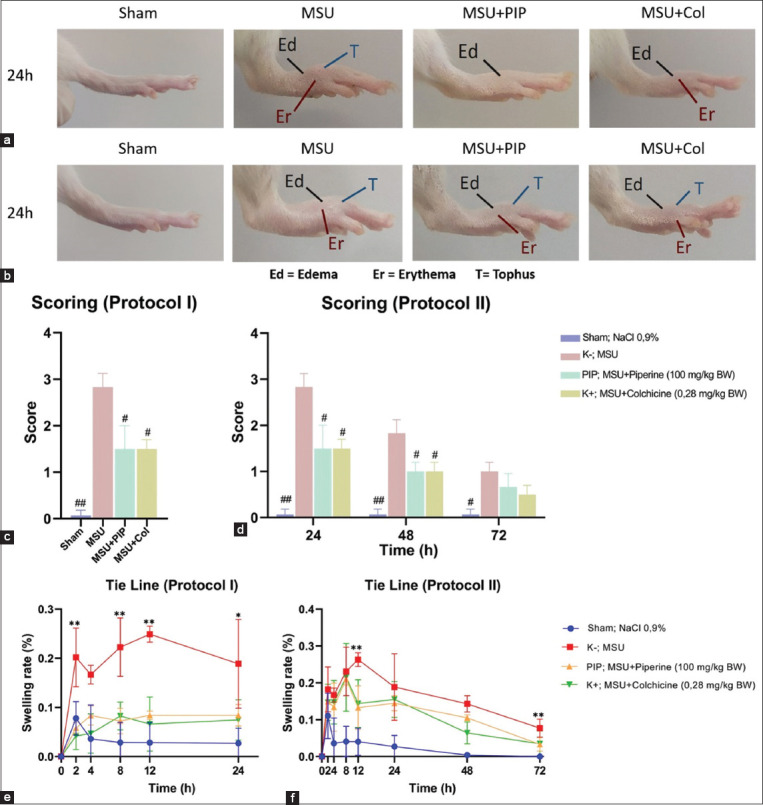
Oral administration of PIP suppresses gouty inflammation induced by injection of monosodium urate (MSU) crystals in rat plantar. (a, c, e) Anatomical assessment of inflammation in preventive protocol I. (b, d, f) Anatomical assessment of inflammation in curative protocol II (a) comparison of rat paw morphology of protocol I 24 h post-injection. (b) Comparison of rat paw morphology of protocol II 24 h post-injection. (c) Protocol I rat plantar inflammation scoring 24 h post-injection. (d) Protocol II rat plantar inflammation scoring 24, 48, and 72 h post-injection. (e) The swelling rate of protocol I rat paws was calculated using the tie line method at 2, 4, 8, 12, and 24 h post-injection. (f) The swelling rate of protocol II rat paws was calculated using the tie line method at 2, 4, 8, 12, 24, 48, and 72 h post-injection. Values on the graph are represented as mean±SD (n=3 rats/group). Significantly different from MSU group, ^#^p<0.05, ^##^p<0.01; significantly different from sham, MSU+PIP, MSU+Col groups, *p<0.05, **p<0.01. MSU=Monosodium urate; PIP=Piperine; Col=Colchicine.

The protocol I 24-h post-injection of rats demonstrated the preventive action of PIP activity. PIP protection against gout was associated with the inhibition of MSU crystal-induced immunopathogenesis (Figure-2a, c, e). Consistent with protocol I PIP immunosuppression, experiments performed using in protocol II group also demonstrated an effect of PIP on the repression of MSU-induced gouty arthritis (Figure-2b, d, f). Morphological analysis of plantar in protocol I rats showed that pre-administration of PIP reduced inflammatory symptoms, including erythema, edema, rapid onset of inflammation within 12 h, and tophus formation in this gout model (Figure-2a). The PIP group also exhibited reduced inflammatory symptoms in protocol II, which indicated a drastic suppressive activity of PIP against MSU-induced inflammation (Figure-2b). Scoring-based quantification of inflammatory symptoms in protocol I 24 h post-injection suggested the amelioration of gout pathogenesis by the preventive activity of PIP (Figure-2c). Scoring in protocol II rats indicated a curative suppression of gout symptoms at all checkpoints (Figure-2d). Experiments to determine the pattern of inhibition of MSU-induced gout by PIP bioactivity were carried out using the tie line method. The swelling rate value (%) represents the formation of edema. Based on the plot on the swelling rate graph for protocol I, PIP protection predominately suppressed the progression and onset of gout at all checkpoints as early as 2 h post-injection (Figure-2e), whereas in protocol II, PIP markedly suppressed inflammation, and edema formation during the pathogenesis of gout at its onset (i.e., 12 h post-injection, Figure-2f). Taken together, the preventive and curative activity of PIP during gout assessment was between that of the sham and MSU groups. In addition, there was a similarity between MSU+PIP and the MSU+Col groups in both protocols for MSU-induced gout inhibition.

### PIP reduces tophus and synovial destruction in MSU-induced gout

**Figure-3:**
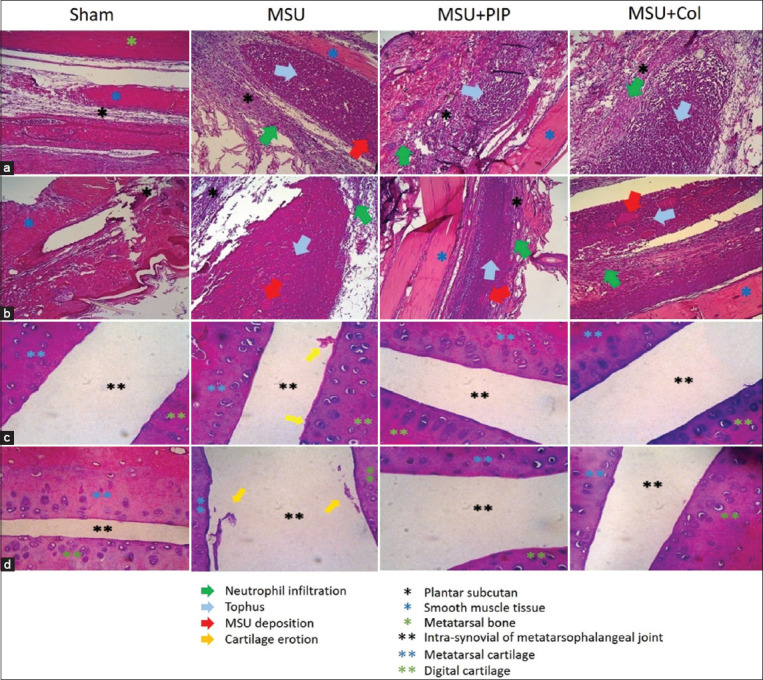
Effects of piperine on the reduction of tophus formation and cartilage erosion in a gout model rat due to intra-plantar injection of monosodium urate. (a and c) Hematoxylin and eosin (H&E) staining of rat plantar in protocol I. (b and d) H&E staining of rat plantar in protocol II. (a) Tophus formation and subcutaneous neutrophil infiltration 24 h post-injection. (b) Tophus formation and subcutaneous neutrophil infiltration 72 h post-injection. (c) Erosion of the metatarsophalangeal cartilage 24 h after injection. (d) Erosion of the metatarsophalangeal cartilage 72 h after injection.

Histopathological microscopic analysis of plantar tissue samples of protocol I and II rats confirmed the potential of PIP as a prophylactic and therapeutic agent for gout by inhibiting acute inflammation, which was demonstrated by an inflammatory assessment. The oral administration of PIP in both protocols demonstrated the immunosuppressive activity of PIP associated with NLRP3 inflammasome inhibition and suppression of the pathogenesis of MSU-induced gout (Figure-3). Comparison of tophus and polymorphonuclear neutrophil (PMN) infiltration in protocol I groups by histopathology 24 h post-injection revealed the preventive effects of PIP, which was able to reduce tophus formation and an influx in plantar subcutaneous tissue in the rats. Pre-administration of PIP also minimized MSU deposits in the tophus center (Figure-3a). The efficacy of PIP for the reduction of tophus gout was also observed in protocol II (Figure-3b). PIP preparations at 100× magnification showed a small-sized tophus and minimal MSU deposition compared with the MSU group. PMN invasion was also dramatically suppressed by PIP. Investigation of the metatarsophalangeal joints at 400× magnification indicated the preventive and curative effects of PIP on cartilage erosion resulting from MSU-induced acute inflammation (Figures-3c and d). Evaluation of protocol I preparations demonstrated the protection of PIP against gout-induced cartilage erosion as observed in the MSU group (Figure-3c). In protocol II group, the proportion of cartilage eroded was higher than that in protocol I. Analysis of protocol II showed the role of PIP in inhibiting cartilage destruction as observed in the MSU group at 72 h post-injection (Figure-3d).

### PIP inhibits leukocyte migration and degeneration in the plantar edema rat gout model

**Figure-4:**
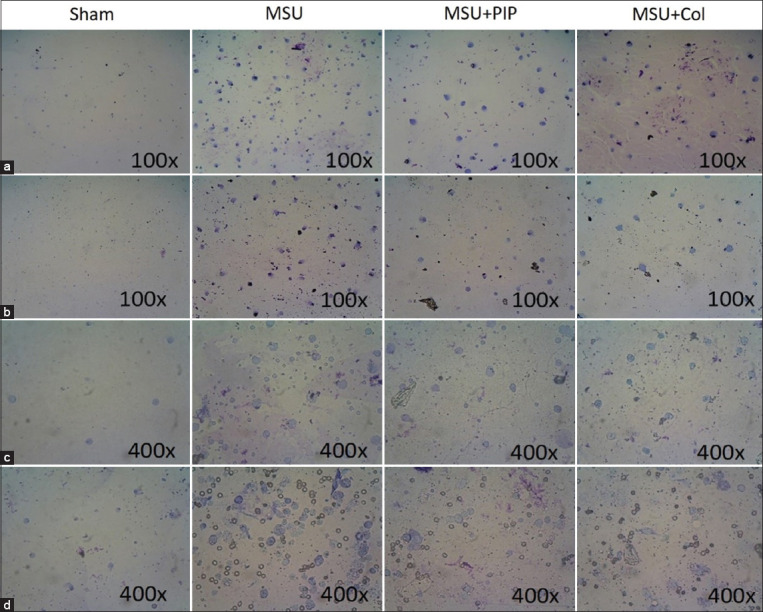
Piperine bioactivity in the inhibition of leukocyte exudation in edema of rat plantar due to monosodium urate injection. (a and c) Giemsa staining of protocol I rat paw exudate. (b and d) Giemsa staining of protocol II rat paw exudate. (a) Resident macrophages and monocytes in edema 24 h post-injection. (b) Resident macrophages and monocytes in edema 72 h after injection. (c) Neutrophil exudation 24 h after injection. (d) Neutrophil exudation 72 h post-injection.

Microscopic analysis of plantar edema in Giemsa-stained rat specimens demonstrated the preventive action of PIP against leukocyte exudation in MSU-induced gout (Figure-4a and c). PIP activity in reducing leukocyte density curatively in gout was also observed in a cytological analysis of edema (Figure-4b and d). Analysis of the plantar edema samples from protocol I rats at 100× magnification indicated that PIP was protective against monocyte-macrophage exudation. This was observed in the PIP protocol I preparations, in which the monocyte-macrophage density was lower compared with that in the MSU group (Figure-4a). Similar results were also observed for edema in protocol II. PIP reduced monocyte-macrophage exudation through its curative action during MSU-induced inflammation (Figure-4b). The neutrophil influx in gouty edema was observed at 400× magnification. Oral pre-administration of PIP is known to inhibit migration and necroinflammation of degenerative neutrophils (Figure-4c). The curative action of PIP was also evident by the relatively lower neutrophil density of the PIP group compared with the MSU group (Figure-4d). A similar pattern in the reduction of leukocyte density was also found in the orally administered Col group. The samples from both protocols demonstrate PIP pharmacodynamics in the inhibition of the NLRP3 inflammasome cascade that suppresses leukocyte migration and degradation in the exudate during MSU-induced gout inflammation.

### PIP inhibits lipid peroxidation in MSU-induced acute gout inflammation

**Figure-5:**
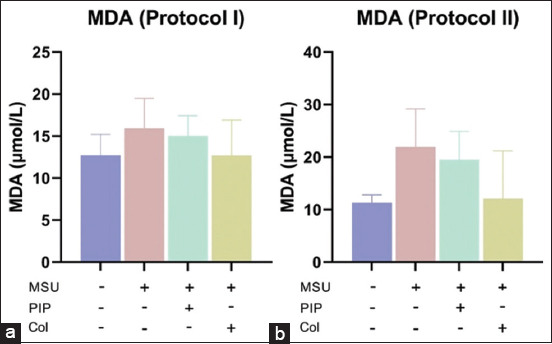
Oral administration of piperine inhibits lipid peroxidation due to monosodium urate-induced gout inflammation, thereby suppressing malondialdehyde (MDA) levels. (a) Serum MDA levels in protocol I. (b) Serum MDA levels in protocol II. Values on the graph are represented as mean±SD (n=3 rats/group).

The quantification of the lipid peroxidation index revealed a decrease in MDA levels in the serum of the PIP-administered gout-afflicted rats (Figure-5). PIP pre-treatment of protocol I rats resulted in protection against elevated serum MDA levels resulting from MSU-induced acute inflammation. PIP inhibition of lipid peroxidation reduced serum MDA levels to a concentration of 15.0 mmol/l (Figure-5a). Evaluation of protocol II MDA levels also indicated a curative action of PIP in the repression of lipid peroxidation from oxidative stress caused by MSU inflammation. PIP activity reduces MDA levels to 19.5 mmol>/l (Figure-5b). Data from both protocols showed that serum MDA levels of rats administered PIP orally were between MSU and MSU+Col.

### PIP reduces hepatic CRP levels resulting from MSU-induced acute inflammation

**Figure-6:**
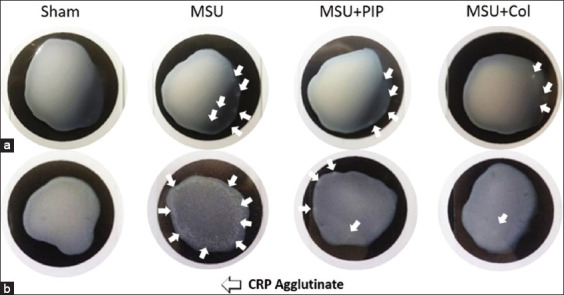
Effect of piperine in reducing C-reactive protein (CRP) in the liver due to monosodium urate-induced inflammation. Detection was carried out based on the agglutination of the 10% liver homogenate supernatant on CRP-latex. (a) Protocol I (preventive) rat liver CRP agglutinate. (b) Rat liver CRP agglutinate protocol II (curative).

Qualitative testing of CRP production by hepatocytes showed a high degree of agglutination in the MSU group compared with the other groups in each protocol. The highest CRP production was observed in the MSU group protocol II samples. This suggests high CRP levels in the liver at 72 h post-injection because of MSU-induced acute inflammation. Pre-administration of PIP in protocol I rats reduced agglutinate density in the rat liver (Figure-6a). Marked PIP inhibition of CRP production was observed in protocol II, in which the agglutinate density of CRP was much lower than that of the MSU group (Figure-6b). Low CRP agglutination was also observed in the MSU+Col group. Analysis of these data indicates the effect of PIP on reducing systemic inflammatory symptoms mediated by CRP reactant in MSU-injected gouty arthritic models. Based on these findings, PIP has the potential to be a prophylactic and therapeutic treatment for the management of the clinical manifestations of gout that are systemic and associated with CRP.

## Discussion

The NLRP3 inflammasome is a multiprotein complex identified as a mediator with an important role in the inflammation of gouty arthritis. It undergoes oligomerization with complex activation following exposure to MSU [24]. The active caspase-1 conformation of the NLRP3 inflammasome results in the conversion of the pro-IL-1β and pro-IL-18 cytokine precursors to their biologically active forms, IL-1β and IL-18. The proteolytic activity of caspase-1 also modulates the secretion of pro-inflammatory cytokines during pyroptosis through the Gasdermin D (GSDMD) N-terminal fragment (GSDMD-N) [25]. Activation of the NLRP3 inflammasome in MSU-induced gout involves two signals. Primary signaling through TLR 2/4-MyD88-IRAK1/4-TRAF6-TAK1-IKK-NF-κB causes upregulation of NLRP3 and pro-IL-1β mRNA expression [11]. The MAPK signaling pathways, including JNK/ERK/p38, activate NF-κB for NLRP3 inflammasome regulation when reactive oxygen species production is induced [13]. In addition to acting on the primary signaling pathway, JNK-1 (MAPK family) also acts on the second activation signal through phosphorylation of Ser198 residues in NLRP3 during post-translational modification and Tyr146 of the ASC adapter during speck aggregation [14,15]. Because of its central role in regulating the NLRP3 inflammasome, the MAPK/NF-κB axis may represent a promising therapeutic target for gout management strategies.

The RMSD value of the PIP ligand <2 is considered accurate [26]. The binding affinity values of PIP to IKK-β and JNK-1 were −5.7428 kcal/mol and −6.0626 kcal/mol, respectively. The smaller the binding affinity value, the higher the affinity between the ligand and the target protein [26]. Visualization and docking analysis can model the competitive inhibition pattern of PIP on the active site of IKK-β and JNK-1. The PIP pharmacodynamic model demonstrates disruption of the MAPK (JNK-1) and NF-κB (IKK-β) signaling pathways, so that they can ameliorate inflammation through the downregulation of the NLRP3 inflammasome.

Gouty arthritis is the most common type of arthropathy and is characterized by inflammation of the joints with MSU as the inducer [1]. Acute inflammation may occur repeatedly during chronic gout and is often referred to as recurrent gout [27]. Metatarsophalangeal articulation is one of the predilections for gout of which patients often complain [2]. Acute inflammation in MSU-induced gout is characterized by a rapid onset, pain, swelling, and erythema [28]. Tophus, which may be palpated as a protrusion in some cases, is an abscess-like cream-textured mass consisting of MSU and degenerative immune cells that can persist and be a predisposition factor for recurrent gout [29]. The rat gout model used in this study was induced by injection of MSU in the plantar of the left foot. MSU is an NLRP3 inflammasome activation factor for the pathogenesis of gout [30]. Col, a first-line drug and an inhibitor of the NLRP3 inflammasome, was used as a reference drug for the *in vivo* experiments [2,31]. Evaluation of the inflammatory assessment in protocol I indicated that PIP is protective and inhibits the initiation of MSU-induced gout. The potential of PIP as a prophylactic agent may have implications for the inhibition of the pathogenesis of recurrent gout. In addition, the protocol I data may also be the basis for a diet containing PIP as protection against gouty arthritis. Protocol II data also demonstrated the potential of PIP administration as an alternative strategy for the therapeutic management of MSU-induced gout.

NLRP3 inflammasome activation by MSU crystal inducers resulting from phagocytosis by macrophages and neutrophils may have implications for the inflammatory pathophysiology of gouty arthritis [32]. The production of inflammatory mediators by leukocytes modulated by the NLRP3 inflammasome auto-amplifies necroinflammation, thereby contributing to the progression of gouty arthritis [29]. MSU deposition can induce the PMN invasion of joints during the initiation phase of gout. In addition, the neutrophil influx was identified as a primary marker of pathological gout [6].

Similar characteristics were also observed in our histopathological studies of protocol I (preventive) and protocol II (curative) of gout induced by intra-plantar injection of MSU crystals (Figure-3). The neutrophil influx in gout model lesions resulting from recruitment by resident macrophages phagocytizes MSU and triggers the release of inflammatory mediators. The secretion of cytokines and chemokines, including pleiotropic TNF-α and IL-1β, modulates vasodilation, erythema, synovitis, tissue destruction, and cartilage erosion [33]. IL-1β secretion stimulates the release of matrix metalloproteinases enzymes, thereby destroying cartilage and bone and involves osteoblasts [34]. Oval or irregular tophus formation was also observed in hematoxylin- and eosin-stained preparations. Tophus was observed as a crystalline mass of MSU and neutrophil extracellular traps (NETs) similar to granulomas and encapsulated CD68+ macrophages, fibroblasts, and giant cells that contribute to the destruction of surrounding tissues [29].

The ability of PIP to reduce the pathogenicity of acute gouty inflammation, including neutrophil influx, tophus formation, and cartilage erosion, is the basis for a PIP-based gout-arthritis treatment strategy. The lack of MSU deposits on the PIP preparations also indicated the potential of PIP to inhibit recurrent gout, considering that MSU is one of the inducers. In addition, the efficacy of PIP makes it a potential candidate for other diseases associated with immunopathogenesis by PMN invasion and leukocytes contributing to acute inflammation.

The initiation of gout occurs because of the interaction between MSU and resident macrophages, which activate the NLRP3 inflammasome complex, a regulator of the pro-inflammatory cytokine, IL-1β [35]. A previous study has reported that IL-1β plays a central role in the initiation of acute gout [36]. Maturation of IL-1β by the NLRP3 inflammasome induces vasodilation, increased edema formation, leukocyte recruitment, and upregulation of the expression of chemokines and pro-inflammatory cytokines. Edema in MSU-induced gout is associated with leukocyte migration, including monocytes, macrophages, and PMNs, during the process of extravasation by activated endothelial cells [8]. This leukocyte exudation is mediated by endothelial expression of immune cell adhesion molecules, such as E-selectin and vascular cell adhesion molecule 1 [24]. Cytology-based studies of *in vivo* gout demonstrated the potential of PIP to inhibit exudation, thereby reducing local edema in MSU-induced gout (Figure-4).

The significant reduction in neutrophil density by PIP also confirmed the ability of PIP to inhibit tophus formation as observed by histopathology in gout samples. These data indicate the preventative and curative activity of PIP for the inhibition of NETosis by neutrophils, which is reported to underlie the formation of aggregated NETs (aggNETs) in tophus [37].

MDA is a marker of lipid peroxidation and it can be used as a parameter in estimating the lipid peroxidation index resulting from oxidative stress during inflammation [28,38]. In addition, there is a correlation between the depletion of anti-oxidant activity and the production of MDA by lipid peroxidation. A previous study has reported increased serum MDA levels produced by monocytes, macrophages, and PMNs in an MSU-induced gouty arthritis model [6].

Gout attacks are often accompanied by a systemic inflammatory response, such as fever, where CRP is known to play a role in the manifestation of systemic symptoms of gout arthritis [39]. CRP is a homopentamer protein found in the acute phase of inflammation and is primarily produced by hepatocytes. This substance is also produced by smooth muscle cells, macrophages, endothelium, lymphocytes, and adipocytes [40]. A previous study has demonstrated the key role of CRP during acute inflammation, which involves complement, apoptosis, phagocytosis, and pro-inflammatory cytokines [41]. The pathogenesis of MSU-induced gout involving these components may be initiated and amplified by IL-1β, a key regulator of gout maturated by the NLRP3 inflammasome [42]. Therefore, PIP is capable of disrupting the NLRP3 inflammasome and has the potential to inhibit CRP production during the acute phase of gout inflammation.

## Conclusion

The data demonstrated the ability of PIP to suppress inflammation in gouty arthritis by downregulating the NLRP3 inflammasome. The molecular docking studies demonstrated the pharmacodynamic model of PIP as a competitive inhibitor of JNK-1 (MAPK family) and IKK-1β (NF-κB activator), thereby disrupting the oligomerization of the NLRP3 inflammasome, which has implications for inflammation inhibition. The *in vivo* experiments demonstrated PIP activity in the suppression of MSU-induced gout in a preventive and curative manner. The results indicate the potential of PIP as a prophylactic and therapeutic agent for gouty arthritis.

## Authors’ Contributions

SIOS: Conceived, supervised, wrote, and revised the manuscript. GAKJ, NA, and AAW: Research investigation, analyzed the data, wrote, and revised the manuscript. All authors read and approved the final manuscript.
